# A Population Pharmacokinetic and Pharmacodynamic Analysis of Peginesatide in Patients with Chronic Kidney Disease on Dialysis

**DOI:** 10.1371/journal.pone.0066422

**Published:** 2013-06-19

**Authors:** Himanshu Naik, Max C. Tsai, Jill Fiedler-Kelly, Ping Qiu, Majid Vakilynejad

**Affiliations:** 1 Pharmacometrics, Takeda Global Research and Development, Inc., Deerfield, Illinois, United States of America; 2 Pharmacometric Services, Cognigen Corporation, Buffalo, New York, United States of America; 3 Clinical Science, Takeda Global Research and Development, Inc., Deerfield, Illinois, United States of America; University of Sao Paulo Medical School, Brazil

## Abstract

Peginesatide (OMONTYS®) is an erythropoiesis-stimulating agent that was indicated in the United States for the treatment of anemia due to chronic kidney disease in adult patients on dialysis prior to its recent marketing withdrawal by the manufacturer. The objective of this analysis was to develop a population pharmacokinetic and pharmacodynamic model to characterize the time-course of peginesatide plasma and hemoglobin concentrations following intravenous and subcutaneous administration. Plasma samples (n = 2,665) from 672 patients with chronic kidney disease (on or not on dialysis) and hemoglobin samples (n = 18,857) from 517 hemodialysis patients (subset of the 672 patients), were used for pharmacokinetic-pharmacodynamic model development in NONMEM VI. The pharmacokinetic profile of peginesatide was best described by a two-compartment model with first-order absorption and saturable elimination. The relationship between peginesatide and hemoglobin plasma concentrations was best characterized by a modified precursor-dependent lifespan indirect response model. The estimate of maximal stimulatory effect of peginesatide on the endogenous production rate of progenitor cells (Emax) was 0.54. The estimate of peginesatide drug concentration required for 50% of maximal response (EC50) estimates was 0.4 µg/mL. Several significant (P<0.005) covariates affected simulated peginesatide exposure by ≤36%. Based upon ≤0.2 g/dL effects on simulated hemoglobin levels, none were considered clinically relevant.

## Introduction

Chronic kidney disease (CKD) is a major public health problem affecting 50 million people worldwide, with recent United States (US) adult prevalence estimates of >13% (>25 million) [1]–[3]. CKD often progresses and may result in end-stage renal disease (ESRD), where the kidneys are no longer functioning and dialysis or kidney transplantation is needed. Among the approximately 570,000 Americans with ESRD during 2009, nearly 400,000 were receiving dialysis [1]–[3].

Erythropoiesis-stimulating agents (ESAs) are considered standard treatment for CKD-related anemia. ESAs provide a stimulatory signal to erythroid progenitor cells located in the bone marrow, thereby treating the anemia and notably reducing the requirement for blood transfusions [4].

Peginesatide (OMONTYS®) is a once-monthly ESA that was recently approved in the US for the treatment of anemia due to CKD in adult patients on dialysis. The compound is a novel, synthetic peptide-based ESA designed and engineered to stimulate specifically the erythropoietin receptor dimer that governs erythropoiesis [4]. It is composed of a dimeric peptide that is linked to a polyethylene glycol (PEG) moiety [4]. The amino acid sequence of peginesatide is unrelated to that of erythropoietin; therefore, peginesatide is unlikely to induce a cross-reactive immune response against either endogenous or recombinant erythropoietin [4]. The pharmacologic and pharmacokinetic (PK) characteristics of peginesatide as a 40-kDa PEG-conjugate, together with its additional functional properties, may contribute to peginesatide’s prolonged erythropoietic action. Peginesatide was voluntarily withdrawn from the market in February 2013 due to the post-marketing reports of serious hypersensitivity reactions, including anaphylaxis observed in some subjects (0.02% after the first intravenous dose).

In patients on dialysis, peginesatide maximum plasma concentration (Cmax) and area-under-the-curve (AUC) increase with dose following single intravenous (IV) or subcutaneous (SC) injection administration at doses ranging from 0.03 to 0.16 mg/kg. The mean (± standard deviation) half-life (T1/2) in dialysis patients is 47.9±16.5 hours following IV administration, with a mean systemic clearance of 0.5±0.2 mL/hr•kg and a mean volume of distribution of 34.9±13.8 mL/kg. Following SC administration to dialysis patients, peginesatide Cmax is reached in approximately 48 hours, with a bioavailability of approximately 46%. No accumulation of peginesatide was observed following IV or SC administration every 4 weeks in dialysis patients [5]. In healthy subjects, similar to other ESAs [6], peginesatide follows flip-flop kinetics and a shorter T1/2 is observed following IV (25.0±7.6 hours) compared to SC administration (53.0±17.7 hours).

The objective of the current analyses was to develop a population PK and pharmacodynamic (PD) model for peginesatide following IV and SC administration in patients with CKD receiving dialysis to characterize the time-course of peginesatide plasma concentrations and hemoglobin levels and to assess the impact of selected covariates in explaining the inter-subject variability associated with the estimation of PK and PD parameters of peginesatide.

## Materials and Methods

### Data and Analyses Utilized for the PK and PK-PD Model Determinations

Data utilized to develop the population PK and PK-PD models for peginesatide were obtained from four Phase 2 studies in CKD patients on and not on dialysis. These studies were: AFX01-02 (NCT00109291) [data on file], AFX01-03 (NCT00228449) [7], AFX01-04 (NCT00228436) [8] and AFX01-07 (NCT00434330) [data on file]). Data were also utilized from one Phase 3 study, AFX01-14 (NCT00597584) [9] conducted in hemodialysis patients. All plasma concentration-time data obtained in Phase 2 (with frequent PK and PD sampling scheme) [7, 8, AFX01-2, AFX01-07] and Phase 3 studies [9], with sparse PK and frequent PD sampling schemes, were used for the development of the PK model. However, only hemoglobin data obtained in studies conducted in hemodialysis patients (AFX01-03 [7], AFX01-07 [data on file], and AFX01-14 [9]) were used for the development of the PK-PD model as peginesatide is currently indicated only for use in treatment of anemia due to CKD in patients receiving dialysis. A review of the peginesatide doses and pharmacokinetic and hemoglobin sampling times implemented in these clinical trials is provided in **Table 1** **.**

**Table 1:** Review of peginesatide doses and pharmacokinetic sampling times, by study.

AFX01-02		AFX01-03^a ^ [^7^]		AFX01-04^b ^ [^8^]		AFX01-07^c^		AFX01-14 [EMERALD 1]^d ^ [^9^]	
**Peginesatide Doses and Cohorts (Number of subjects per cohort)**									
Cohort (Number in PK substudy)	Single dose	Cohort (Number in PK substudy)	Dose Q 4 W for 6 doses	Cohort (Number in PK substudy)	Starting dose and regimen	Cohort (Number in PK substudy)	Starting Dose and Regimen	Study population	Starting Dose
1 (7)	0.05 mg/kg IV	1 (15)	0.033 mg/kg IV	1 (9)	0.05 mg/kg q 4 W SC	1 (3)	0.04 mg/kg IV q4w	549 subjects	0.04 mg/kg to 0.16 mg/kg (based on patient’s prior erythropoietin dose) IV or SC Q 4 Weeks
2 (4)	0.025 mg/kg IV	2 (15)	0.041 mg/kg IV	2 (4)	0.05 mg/kg q 4 W SC	2 (4)	0.05 mg/kg IV q4w		
3 (2)	0.1 mg/kg IV	3 (15)	0.050 mg/kg IV	3 (6)	0.075 mg/kg q 4 W SC	3 (1)	0.075 mg/kg IV q4w		
		4 (15)	0.050 mg/kg IV	4 (5)	0.025 mg/kg q 4 W SC	4 (1)	0.10 mg/kg IV q4w		
		5 (15)	0.066 mg/kg IV	5 (5)	0.05 mg/kg q 4 W IV	5 (4)	0.04 mg/kg SC q4w		
		6–8 (15 per cohort)	Weight-based dosing of 0.05 to 0.15 mg/kg IV	6 (4)	0.025 mg/kg q 2 W SC	6 (5)	0.05 mg/kg SC q4w		
		9 (15 subjects)	0.05 mg/kg IV	7 (0)	0.0375 mg/kg Q 2 W SC	7 (3)	0.075 mg/kg SC q4w		
		10 -11 (15 per cohort)	Fixed dose of 4.0, 6.0, 12.0, or 16.0 mg IV	8 (2)	0.05 mg/kg Q 4 W SC	8 (2)	0.10 mg/kg SC q4w		
				9 (7)	4.0 mg fixed dose Q 4 W SC				
				10 (11 subjects)	3.0 mg fixed dose Q 4 W SC				
**Pharmacokinetic Sample Collection Schedules**									
PK sampling on days 1 and 2: Samples drawn pre-dose and after the start of study drug administration at 5 and 15 minutes, and at hours 1, 4, 8 and 24 hours		Prior to Dose 1 and post-Dose 1 at 5, 15, and 60 minutes, and at 4 and 24 hours, pre- and post- the next dialysis (approximately 44 and 48 hours), at 96 hours (±24 hours) and after 1 week		Pre-dose (prior to Dose 1) and post-Dose 1 at 2 hours (±10 minutes), and at Days 1 (24±1 hour), 2 (48±2 hours), 4 (96±4 hours), 6 (144±6 hours), 7 (168±6 hours) and 9 (216±6 hours)		IV Patients Post-Day 1 Dose. Pre-dose and post-dose at 5 and 15 minutes, and at hours 1, 4 (±10 minutes), 24 (±1 hour), 44, 48, 96 (±4 hours), and 168 (±6 hours) post-dose		Blood samples for population PK analysis were collected at Week 1 prior to Dose 1 and approximately 48 to 72 hours post-Dose 1 of peginesatide injection	
		PK sampling on Week 5 and 9 dosing days: Samples drawn pre-dose and post-dose at 15 minutes		PK sampling at Weeks 5, 9, 13, 17 and 21 dosing days: Samples drawn pre-dose only		SC Patients Post-Day 1 Dose. Pre-dose and post-dose at hours 2 (±10 minutes), 24 (±1 hour), 48 (±2 hours), 96 (±4 hours), 144 (±6 hours), 168 (±6 hours), and 216 (±6 hours)			
		PK sampling on Week 13, 17, and 21 dosing days: Samples drawn pre-dose only		Optional PK profile drawn from Dose 2 onwards. Samples will be drawn prior to dosing and post-dosing at 2 hours (±10 minutes), and at Days 1 (24±1 hour), 2 (48±2 hours), 4 (96±4 hours), 6 (144±6 hours) 7 (168±6 hours), and 9 (216±6 hours) after that dose		PK Sampling for Subsequent Doses: Samples drawn pre-dose only			
Hemoglobin Measurement Times for PD Analysis									
Not included in PD analysis		Baseline and Screening: Tree mid- or end-of-week hemoglobin values		Not included in PD analysis		Baseline and Screening: Three mid- or end-of-week hemoglobin values		Baseline and Screening: Hemoglobin at screening and at baseline before randomization	
		Weekly Visit: Mid- or end-of-week hemoglobin values				Week 1:Mid- or end-of-week hemoglobin values		Titration Period: Hemoglobin every 2 weeks ±3 days except in the case of a dose delay. During a dose delay, hemoglobin must be assessed weekly until dosing is resumed	
		Study Termination: Hemoglobin levels at termination visit				Weekly Visit (±2 days); Mid- or end-of-week hemoglobin values		Evaluation Period: Hemoglobin determined weekly	
						Study Termination: Mid- or end-of-week hemoglobin values		Efficacy Period: Hemoglobin every 2 weeks ±3 days except in the case of a dose delay. During a dose delay, hemoglobin must be assessed weekly until dosing is resumed	

Peginesatide concentrations in plasma were measured using a validated enzyme linked immunosorbent assay (ELISA) with a calibration curve that ranged from 11.72 to 1500 ng/mL and anchor points at 11.72 and 1500 ng/mL. The lower and upper limits of quantification for the assay were 25 and 600 ng/mL, respectively. The inter-day analysis of quality control (QC) plasma samples for peginesatide (25, 50, 100, 450 and 600 ng/mL) demonstrated precision of the bioanalytical method with relative standard deviation ranging from 0.7 to 22.8% (n = 18). Inter-day accuracy for the plasma QC samples for peginesatide ranged from -21.3 to 17.8% (n = 18).

The concentration of hemoglobin was measured using a standard laboratory method in a Clinical Laboratory Improvement Amendments certified central laboratory. Baseline weight was used to calculate the dose (in mg/kg). The first order conditional estimation (FOCE) and first-order (FO) method as implemented in NONMEM were used for PK and PK-PD modeling. Model selection criteria included a statistically significant reduction of the objective function value (OFV) (α = 0.05) for nested models, Akaike information criteria for non-nested models, reductions in both residual (σ^2^) and inter-subject (ω^2^) variability, improvement in the precision of parameter estimates as measured by the percent standard error of the mean (%SEM), and visual examination of standard goodness-of-fit plots.

### Analysis Software

NONMEM software version VI, level 1.0 (ICON Development Solutions, Ellicott City, Maryland, US) was used for population PK-PD modeling [10]. Graphical plots were generated using S-PLUS version 8.0 (Tibco Software Inc, Palo Alto, CA), Microsoft Excel 2007 (Microsoft Corp, Redmond, Washington, US), R®version 2.12.1 (The R Foundation for Statistical Computing, Vienna, Austria), SAS® version 9.2 (2009; SAS Institute, Cary, North Carolina, US), and Perl Speaks NONMEM (Uppsala, Sweden).

### PK and PK-PD Model Development

Several different PK models were evaluated as base structural PK models. These included: 1-compartment and 2-compartment with first-order absorption and elimination; 1-compartment and 2-compartment with first-order absorption and Michaelis-Menton (MM) elimination; and a combination of zero- and first-order absorption with linear and MM elimination. Life-span, negative-feedback and precursor-dependent Lifespan Indirect Response (LIDR) models were evaluated as potential base PK-PD models.

**Figure 1:**
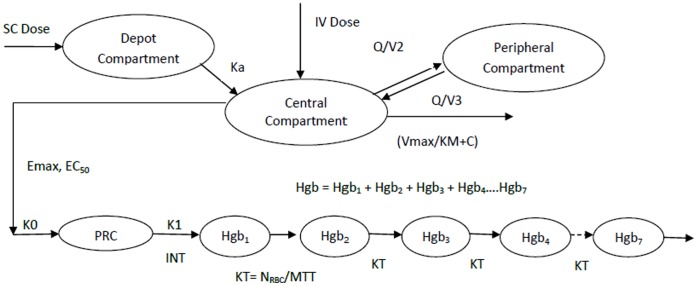
Schematic of two-compartment, precursor-dependent, lifespan indirect response population PK/PD model for peginesatide following subcutaneous or intravenous administration. Abbreviations: SC = subcutaneous; Ka = Absorption rate constant (1/hr); IV = intravenous; Q = Apparent distribution clearance from the central to the peripheral compartment (mL/kg/hr); V3 =  Peripheral volume of distribution; V2 =  Central volume of distribution; Vmax = Maximum rate of elimination (ng/mL/hr); KM = Concentration needed to reach 50% of Vmax (ng/mL); C = Peginesatide serum concentration (ng/mL); Hgb = Hemoglobin (g/dL); Hgb_1–7_ =  Transit Compartment; Emax = Maximum stimulatory effect of peginesatide on progenitor cell production; EC_50_ =  Peginesatide serum concentration necessary to stimulate progenitor cell production rate at half of the maximum response (ng/mL); K0 =  Endogenous production rate constant of progenitor cells; PRC = Precursor cell compartment; K1 =  First-order transition rate constant from precursor cell to red blood cell; KT = First-order rate constant between the aging compartments; N_RBC_/MTT = Life span of red blood cells.

A sequential PK-PD modeling approach was used to characterize the time-course of peginesatide plasma concentrations (CP) and hemoglobin levels following IV and SC peginesatide administration to hemodialysis patients. A modified precursor-dependent LIDR model with stimulation of the production rate of precursor cells as described in **Equation 1** and **Figure 1** was used as the base PK-PD model:
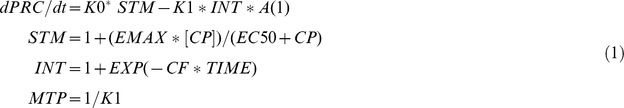
Where:

dPRC/dt = Rate of change in production of precursor cells over time,

K0 =  Endogenous production rate constant for progenitor cells,

STM = Stimulation factor,

K1 =  First-order transition rate constant from precursor cell to red blood cells,

INT = Exponential function to empirically account for the downward shift in hemoglobin levels during trial,

A(1) = The amount of precursor cells,

EMAX = Maximal stimulatory effect of peginesatide on K0,

CP = Plasma concentration of peginesatide,

EC50 =  Peginesatide concentration required for 50% of maximal response,

CF = Correction factor, and.

MTP = Mean transit time for progenitor cells.

The model used to describe the relation between peginesatide concentration and hemoglobin levels was based upon that used for darbepoetin, with slight modifications [6]. In order to account for the life span of red blood cells (RBC) in describing the time course of hemoglobin, a series of compartments linked together by first order cell transfer rates was incorporated in the model as shown in **Figure 1**. Each compartment represents a pool of red blood cells of increased mean age by 1/KT where KT = First order rate constant between the aging compartments [6]. A cascade of N_RBC_ = 7 age compartments with the transfer rate constants equal to N_RBC_/MTT was selected to account for the hemoglobin in the RBC, where MTT is the mean RBC life span. The precursor cells in bone marrow are related to the blood as the youngest circulating reticulocytes (red blood cells), according to a first-order rate, K1, following Equation 2:
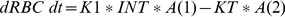
(2)Where:

dRBC/dt = Rate of change in production of RBC over time,

INT = Exponential function to empirically account for the downward shift in hemoglobin levels during trial,

A(1) = The amount of precursor cells,

KT = First-order rate constant between the aging compartments, and.

A(2) = Amount of red blood cells.

And then they mature to red blood cells through a series of age compartments as in Equation 3 [6]:
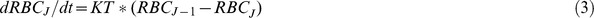
Where:

J = 2, 3,….N_RBC_.

Therefore, circulating red blood cells (RBC) are the sum of red blood cell reticulocytes at all ages, as in Equation 4 [6]:
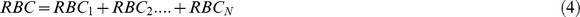



As hemoglobin production is directly proportional to the production of RBCs, then Equation 4 (as above) can be transformed as follows (Equation 5 [6]):
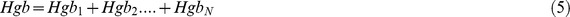
Where:

Hgb = observed hemoglobin levels.

Therefore, the red blood cell life span is equivalent to hemoglobin life span, and the initial conditions for the production rate of precursor cells and red blood cells (or hemoglobin) can be determined from the baseline hemoglobin as in Equations 6 and 7:
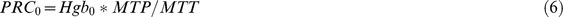


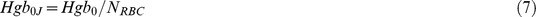
Where:

PRC_0_ =  Precursor cells at baseline,

Hgb_0_ =  Observed baseline hemoglobin, and.

MTT = Mean red blood cell life span.

The endogenous progenitor production rate constant at baseline was calculated from Equation 1 as:
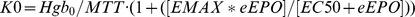
Where:

eEPO = Endogenous erythropoietin for each individual at the start of the trial.

In the study, baseline erythropoietin levels were not measured in all patients. Instead of using actual baseline erythropoietin values to calculate the endogenous progenitor production rate constant at baseline, the values were estimated in the form of the residual effect of previous ESAs (RSA).

Thus the effect of previous ESAs at baseline was calculated using Equation 8 at steady state:
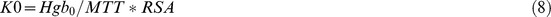
Where:



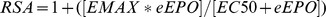



### Covariate Screening, Assessment and Analyses

Initially all plausible covariates and model parameter relationships were explored graphically. The clinical relevance and biological plausibility of covariate-parameter relationships were considered and discussed after development of the final model (**Table 2** **and** **Table 3**). In addition, concomitant medications that were taken by more than 10% of the patient population during 80% of the trial period were also evaluated (**Table 2**).

**Table 2:** Summary of categorical covariates and concomitant medications, by population group.

	PK Population (N = 672)			PK-PD Population (N = 517)			
Variable and Categorical Covariate	Number	Percentage	Number		Percentage		
**Gender**							
Male	408	60.7	318			61.5	
Female	264	39.3	199			38.5	
**Race**							
Black	250	37.2	167			32.3	
White	384	57.1	325			62.9	
Asian	24	3.6	15			2.9	
Other	14	2.1					
**Ethnicity**							
Hispanic	99	14.7	85			16.5	
Non-hispanic	573	85.3	432			83.5	
**Subject** **Type**							
Dialysis patient	602	89.6	517			-	
Non-dialysis patient	70	10.4	-			-	
Beta-blocker at BL	280	51.0	235			50.5	
Intravenous Iron at BL	331	60.3	287			61.7	
Congestive heart failure	208	37.9	174			37.4	
Arrhythmia	85	15.5	74			15.9	
**Subjects on Concomitant Medication During Study**							
ACE Inhibitors	188	28.0	130			25.1	
Antidiabetics	78	11.6	57			11.0	
Angiotensin II receptor blockers	147	21.9	114			22.1	
Aspirin	277	41.2	223			43.1	
Beta-blockers	315	46.9	239			46.2	
Calcium channel blockers	260	38.7	185			35.8	
Diuretic	124	18.5	73			14.1	
Folic acid	122	18.2	100			19.3	
Heparin	454	67.6	388			75.1	
Insulin	139	20.7	100			19.3	
Iron supplement	177	26.3	140			27.1	
Other antihypertensives	260	38.7	226			43.7	
Phosphate binder	476	70.8	395			76.4	
Statin	228	33.9	157			30.4	
Vitamin(s)	506	75.3	426			82.4	
Nonsteroidal anti-inflammatory drugs	47	7.0	32			6.2	
Warfarin	26	3.9	21			4.1	
Antiplatelet agents	60	8.9	53			10.3	

**Table 3:** Patient demographics and laboratory characteristics at baseline, by population group.

Characteristic/Parameter, units	PK Population^1^					PK-PD Population^2^						
	Number	Mean (SD)		Range		Number			Mean (SD)		Range	
**Demographic Parameters**												
Age, in years	672	58.4 (14.6)	21.0–93.0		517		57.9 (14.6)			22.0–93.0		
Body Mass Index, kg/m^2^	669	27.9 (6.8)		14.8–62.8		517		27.6 (6.9)			14.8––62.8	
Weight, in kg	672	79.4 (21.6)		38.0–187.5		517		78.7 (21.6)			38.0–187.5	
**Laboratory Characteristics**												
Albumin, in g/L	607	38.9 (3.8)		25.2–49.0		465		38.7 (3.5)			25.2–47.0	
ALP, in U/L	659	111.1 (96.9)		28.0–1573.2		517		113.9 (105.9)			31.8–1573.2	
ALT, in U/L	659	17.3 (32.7)		1.2–636.0		NR		NR			NR	
AST, in U/L	659	17.3 (15.0)		1.8–235.8		517		16.8 (16.4)			1.8–235.8	
Serum creatinine, in mg/dL	660	8.7 (3.3)		1.3–19.4		517		9.4 (2.9)			1.4–19.4	
Creatinine clearance, in mL/min	660	12.8 (10.0)		3.4–106.3		517		10.6 (6.2)			3.4–78.1	
TBILI, in g/L	659	9.1 (4.0)		2.0–38.0		517		9.5 (4.1)			2.6–38.0	
Calcium, in mmol/L	606	2.2 (0.2)		0.8–3.0		465		2.3 (0.2)			0.8–3.0	
Ferritin, in ng/mL	665	701.9 (718.0)		12.0–14477.0		517		757.9 (778.6)			12.0–14477.0	
eGFR, in mL/min	660	8.4 (7.4)		2.6–70.1		571		6.7 (4.0)			2.6–53.7	
Erythropoietin dose at baseline, in units per week	NR	NR		NR		516		11029.9 (11865.6)			954.0–105910.0	
Hemoglobin, in g/L	665	110.7 (9.1)		75.0–152.0		517		111.8 (8.4)			75.0–152.0	
Potassium, in mmol/L	658	5.1 (0.9)		2.8–8.8		517		5.2 (0.9)			2.8–8.8	
Platelets, in 10^∧^9 L	634	216.5 (73.2)		39.0–577.0		517		214.6 (70.1)			39.0–577.0	
Total Prothrombin, in gm/dL	607	70.5 (5.9)		51.0–91.0		NR		NR			NR	
Total Protein, in gm/L	NR	NR		NR		465		70.7 (5.6)			51.0–91.0	
Red blood cells, in 10^∧^12L	665	3.6 (0.4)		2.6–5.2		517		3.7 (0.4)			2.6–5.2	
Hematocrit, as percentage	655	34.8 (3.7)		24.8–60.1		517		35.5 (3.4)			25.6–60.1	
Reticulocyte Count, as percentage	123	57.9 (28.6)		10.0–170.0		52		68.3 (35.0)			10.0–170.0	
White blood cells, in 10^∧^9 L	655	6.7 (2.2)		2.1–18.1		517		6.8 (2.2)			2.1–18.1	
Transferrin Saturation	663	0.3 (0.1)		0.1–1.0		517		0.3 (0.1)			0.1–1.0	
Dialysis adequacy, in Kt/V	440	1.6 (0.5)		0.1–7.7		495		1.6 (0.4)			0.1–7.7	
C–reactive protein, in nmol/L	534	99.3 (157.1)		1.9–1497.2		463		94.3 (125.0)			1.9–1104.8	

1Total of 672 subjects contributed a total of 2665 samples.

2Total of 517 subjects contributed a total of 18857 samples.

ALP = alkaline phosphate; SGPT or ALT = alanine aminotransferase; SGOT or AST = aspartate aminotransferase; TBILI = total bilirubin; eGFR = estimated glomerular filtration rate; NR = not reported; Kt/V = dialyzer clearance of urea (K) • dialysis time over volume of distribution of urea, approximately equal to patient’s total body water.

A generalized additive modeling (GAM) approach along with graphical analysis (plots of the inter-subject error terms for each parameter [ηi] versus covariates) was used for initial covariate screening and to determine the functional form of the relationship between the covariates and PK and PD parameters of peginesatide. In order to avoid possible multicollinearity issues, GAM analyses were performed for PK and PD parameters using different combinations of covariates that were deemed to be highly correlated (r>0.6) along with the remaining covariates. The potential covariates selected following GAM analysis were then tested using a standard approach of stepwise forward addition (*P*<0.05) followed by stepwise backward elimination procedure (P<0.005). The list of covariates evaluated for PK and PD parameters of peginesatide in GAM and NONMEM is presented in **Table 4** and **Table 5**, respectively. The relationship between continuous covariates and PK and PD parameters were evaluated using linear, exponential and power functions as described in Equations 9, 10, and 11 with the covariates centered or scaled at their median values:
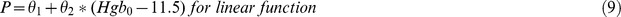


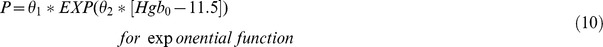


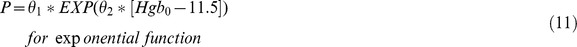



**Table 4:** Covariates evaluated on the PK and PD parameters of peginesatide.

Covariate	PK Parameters	PD Parameters	Covariate	PK Parameters	PD Parameters
Age	Yes	Yes	Beta blocker	Yes	Yes
Gender	Yes	Yes	Calcium channel	Yes	Yes
RAC 1(Black)	Yes	Yes	Insulin	Yes	Yes
RAC 2 (Caucasian)	Yes	Yes	Statin	Yes	Yes
RAC 3 (Asian)	Yes	Yes	Diuretic	Yes	Yes
Ethnicity	Yes	Yes	Phosphate binder	Yes	Yes
Weight (WT)	Yes	Yes	Warfarin	No	Yes
Body mass Index (BMI)	Yes	Yes	ACE inhibitor	Yes	Yes
eGFR	Yes	Yes	Antidiabetic	Yes	Yes
Creatinine (CR)	Yes	Yes	ARBs	Yes	Yes
Hgb	No	Yes	Vitamin	Yes	Yes
RBC	No	Yes	Folic acid	Yes	Yes
Reticulocytes	No	Yes	Other hypertensive	Yes	Yes
Albumin	Yes	Yes	Aspirin	Yes	Yes
ALP	Yes	Yes	Iron supplement	Yes	Yes
AST	Yes	Yes	Heparin	Yes	Yes
TBILI	Yes	Yes	Antiplatelet	Yes	Yes
CA	No	Yes			
Creatinine Clearance (Ccr)	Yes	Yes			
Ferritin(FERR)	No	Yes			
Total protein	No	Yes			
Hematocrit (HT)	No	Yes			
Potassium (K)	Yes	Yes			
Platelets	No	Yes			
ESAD	Yes	Yes			
WBC	No	Yes			
Transferrin	No	Yes			
Dialysis adequacy (Ktv)	No	Yes			
C-reactive Protein	No	Yes			
Arrhythmias (CVARR2N)	No	Yes			
Congestive heart failure(CHFN)	No	Yes			
Phosphate level at baseline (PHBL)	No	Yes			
Beta blocker at baseline (BBBLN)	No	Yes			
IV Iron at baseline (IVIRONBN)	No	Yes			
Patient Type (Patient on dialysis vs Not on dialysis)	Yes	No			

**Table 5:** Covariates evaluated on the PK and PD parameters of peginesatide in NONMEM using forward selection and backward elimination approach.

Parameter	Significant Covariates
**Pharmacokinetic Parameters**	
V2	Age, BMI, ALP, SGOT, eGFR, TBIL, ESAD, Ethnicity, Patient type
KM	WTKG, ALP, K, ESAD, Patient type
Ka	BMI, CR, TBIL, Ethnicity, Patient type
V2	Anti Diabetic, Aspirin, Other Hypertensive agents, Statin, Patient type
KM	Beta Blockers, Other Hypertensive agents, Statin, Patient type
**Pharmacodynamic Parameters**	
BL	HT,AGE,K,FERR,TRSA,ESAD,RACE,TPRO, Anti-viral, statin, IV iron
CF	HT,AGE,FERR,ESAD, ACE Inhibitor, antiviral, Statin, IV iron, heparin, insulin
RSA	Ccr, Anti-diabetic, iron supplement, warfarin, CVARR2, CHF, PHBL
EC50	BMI, TPRO, ESAD, HT, vitamin, warfarin, CVARR2, PHBL, BBBL

Where:
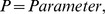


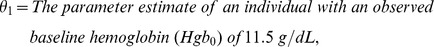


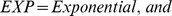


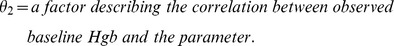



The influences of binary covariates on the parameter were modeled using an additive relationship as described in Equation 12 below:
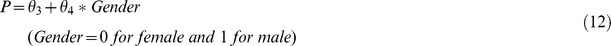



Where:
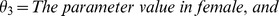


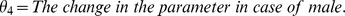



To evaluate the clinical significance of the effect of statistically significant PK and PD covariates, the final population PK and PK-PD models developed were used to simulate exposure and hemoglobin levels following administration of peginesatide 10 mg SC every 4 weeks. This 10 mg dose (equivalent to 0.12 mg/kg dose based upon a mean weight of 79 kg) was used for the simulations since it was in the range of clinical relevant doses administered in the AFX01-14 recently published Phase 3 trial [9].

### Model Evaluation

The adequacy of the final PK and PK-PD models was evaluated using a simulation-based prediction-corrected (pc) visual predictive check (VPC) method (1000 replicates). Due to the dose adjustment algorithm used in these trials, the pcVPC was utilized. This technique provides an enhanced ability to diagnose possible model misspecification by removing the variability introduced in an ordinary VPC when binning across a potentially large variability in dose or other influential covariates [11]. The percent of observations outside the 90% prediction interval were also calculated to evaluate predictive ability of the model. A non-parametric bootstrap procedure (1000 bootstrap runs) was also employed to evaluate the precision of the parameter estimates and the robustness of the final PK and PK-PD models.

## Results

A total of 2,665 peginesatide plasma samples collected from 672 CKD patients on or not on dialysis enrolled in five clinical trials (one phase 3 trial: AFX01-14 [9] and four Phase 2 trials [AFX01-02 [data on file], AFX01-03 [7], AFX01-04 [8], AFX01-07 [data on file]]) were used in the population PK analysis. A total of 18,857 hemoglobin concentrations collected from 517 hemodialysis patients enrolled in two of the Phase 2 trials (AFX01-03 [7], AFX01-07 [data on file]) and the one Phase 3 trial (AFX01-14 [9]) were used for the PK-PD analysis. In 63 subjects, hemoglobin concentration data collected during the time of transfusion (n = 237 samples) or phlebotomy (n = 48 samples), or while they experienced gastrointestinal bleeding (n = 15 samples) or trauma (n = 12 samples) were excluded from the PK-PD analysis. Demographics and baseline characteristics for these two analysis populations are shown in **Table 2** and **Table 3**.

### Pharmacokinetic Model of Peginesatide

**Figure 2:**
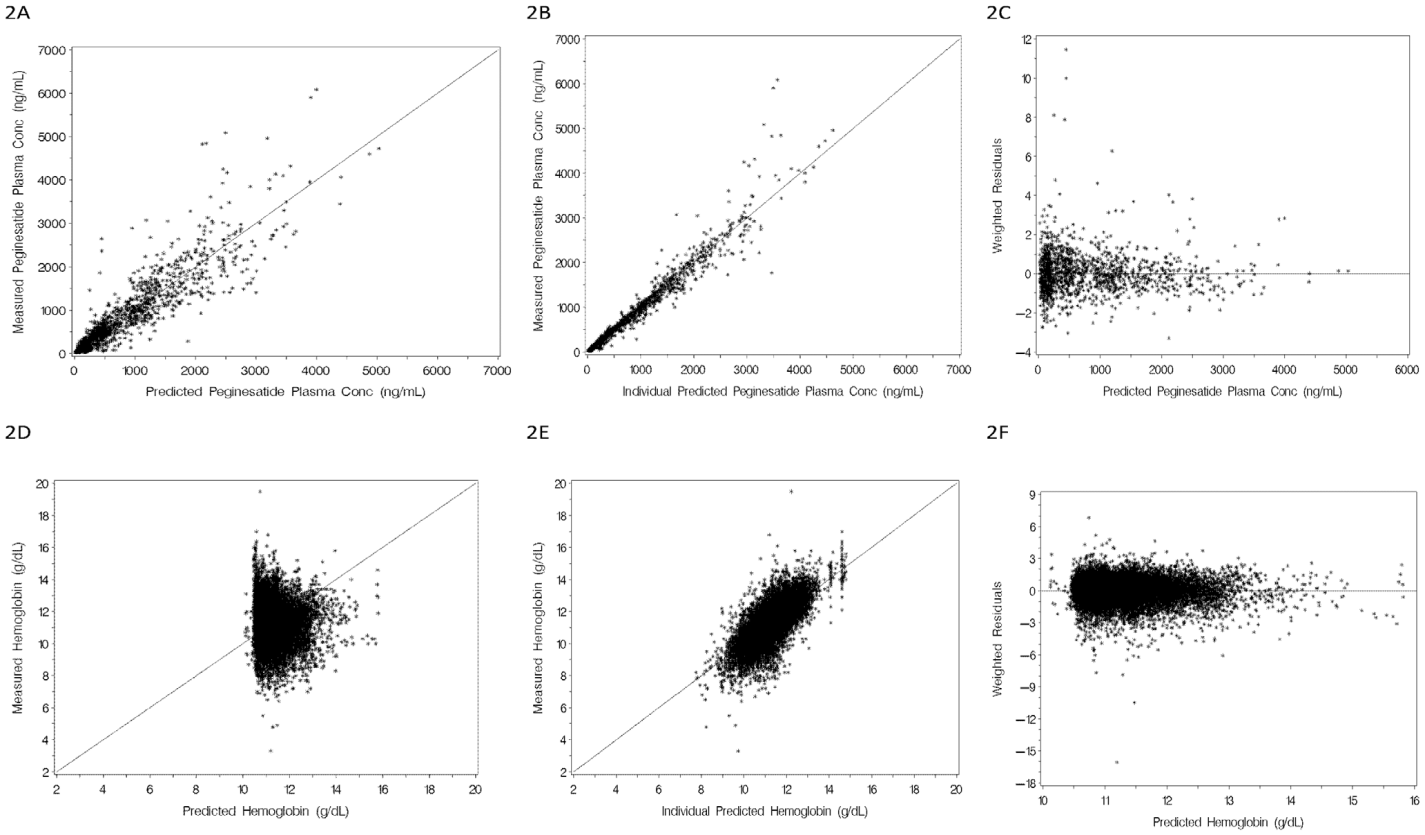
Diagnostic goodness-of-fit plots for the final population PK model of peginesatide (2A-C:upper panel set) and for the final population PK-PD model of peginesatide (2D-F: lower panel set). Upper left (2A): measured concentration versus predicted concentration; Upper middle (2B): measured concentration versus individual predicted concentration; Upper right (2C): weighted residuals versus predicted concentration. Lower left (2D): measured hemoglobin versus predicted hemoglobin; Lower middle (2E): measured hemoglobin versus individual predicted hemoglobin; lower right (2F): weighted residual versus predicted hemoglobin concentration.

Peginesatide PK administered either as an SC or IV dose in CKD patients on and not on dialysis is best described by a two-compartment model with first-order absorption and saturable MM elimination, where inter-compartmental clearance was fixed to improve model stability (**Figure 1**). As summarized in **Table 6** **,** most of the PK model parameters were estimated with good precision and, as illustrated in **Figure 2**, no noticeable trends or biases in the diagnostic goodness-of-fit plots were observed. Inter-individual variability (IIV) was estimated for volume of the central compartment (V2), the absorption rate constant (Ka) and the concentration required to reach 50% of maximum rate of elimination (KM) using exponential models, assuming a log-normal distribution for each parameter. The residual variability was best described using a combination of additive and constant coefficient of variation (CCV) error model.

**Table 6:** Peginesatide parameter estimates and their associated precision for the final PK model, including bootstrap evaluation results.

Parameter	Final Estimate (% SEM)	Median Bootstrap Estimate	Bootstrap 95% CI^1^	
Maximum rate of elimination (Vmax), in ng/mL/hr	45.3 (10.0)	44.7	32.0–69.0	
Concentration needed to reach 50% of Vmax (KM),in ng/mL	1880 (14.6)	1860	1120.0–3222.5	
Central volume of distribution (V2), in mL/kg	35.6 (2.7)	35.6	33.3–38.0	
Absorption rate constant (Ka), in 1/hr	0.00865 (15.6)	0.00869	0.00582–0.013	
Subcutaneous bioavailability (F1)	0.498 (4.4)	0.499	0.430–0.577	
Inter–compartmental clearance (Q), in mL/kg/hr	5.23	5.23	Fixed	
Peripheral volume of distribution (V3), in mL/kg	7.44 (10.3)	7.42	5.49–9.84	
BMI power for V2	–0.491 (8.7)	–0.485	–0.685 to –0.280	
Age slope for V2, in L/yr	–0.125 (19.0)	–0.125	–0.238 to –0.0015	
ALP power for KM	–0.194 (25.5)	–0.190	–0.307 to –0.0895	
TBILI slope for V2, in L/(g/L)	0.477 (22.4)	0.482	0.086–0.933	
Serum creatinine slope on Ka (where PDIA = 1)^2^,in (1/hr)/(mg/dL)	7.84E–04 (69.1)	6.9E–04	–13E–04 to 23E–04	
ETHN shift for Ka, in (1/hr)	0.00811 (20.3)	0.00815	0.0036–0.0117	
ω^2^ on KM	0.0589 (29.0)	0.0575	0.032–0.102	
ω^2^ on V2	0.101 (8.1)	0.100	0.0646–0.1372	
Cov between V2 and Ka	–0.0928 (25.4)	–0.0904	–0.136 to –0.0342	
ω^2^ on Ka	0.197 (32.0)	0.190	0.0964–0.3391	
Ratio of proportional to additive residual variability	0.0218 (19.5)	0.0223	0.0139–5.15	
σ^2^ (additive component)	81.8 (36.8)	78.2	0.0017–166.7	
IIV in KM	24.3%	NA	NA	
IIV in V2	31.8%	NA	NA	
IIV in Ka	44.4%	NA	NA	

1.Based on 870/1000 successfully converged bootstrap runs.

2.PDIA = 1 for patients not on dialysis (covariate was considered only for those not on dialysis).

Following covariate analysis, ethnicity (ETHN) and serum creatinine (CR, only for non-dialysis patients) for Ka; total bilirubin (TBIL), age, and body mass index (BMI) for V2; and alkaline phosphatase (ALP) for KM were identified as statistically significant (P<0.005) covariates (Equations 13, 14 and 15). The inclusion of statistically significant covariates in the PK model reduced the IIV for Ka, V2, and KM by 22.6%, 1.5%, and 13.4%, respectively.
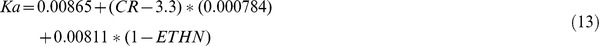


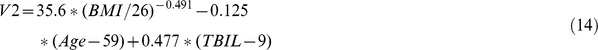


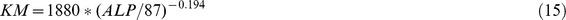



### PK-PD Model of Peginesatide

The time-course of peginesatide plasma concentrations and hemoglobin levels was well characterized by a modified precursor-dependent LIDR model, as presented in **Figure 1**. Most PK-PD model parameters were estimated with good precision (**Table 7**) and no noticeable trends or biases (**Figure 2**) in diagnostics plots (except for the plot of observed versus population predicted hemoglobin concentration). IIV was estimated as an exponential model for drug concentration required for 50% of maximum response (EC50), predicted baseline hemoglobin (HgbBL), residual effect of previous ESAs (RSA) and correction factor (CF), a parameter empirically accounting for the downward shift in hemoglobin levels observed over the time course of peginesatide treatment. The residual variability was best described using an additive error model (equivalent to proportional error model for non log-transformed hemoglobin concentrations).

**Table 7:** Peginesatide parameter estimates and their associated precision for the final PK-PD Model, including bootstrap evaluation results.

Parameter	Final Estimate (% SEM)	Median Bootstrap Estimate	Bootstrap 95% CI^1^
Drug concentration required for 50% of maximumresponse (EC_50_), in ng/mL	401 (2.0)	417	128–12420
Maximum pharmacologic effect (Emax)	0.542 (1.6)	0.564	0.3254–2.976
Hemoglobin at baseline, in g/dL	11.5 (0.4)	11.5	11.4–11.5
Mean transit time for red blood cells (MTT),in hours	1640 (0.5)	1610	1330–1866
Mean transit time for progenitor cells (MTP),in hours	462 (1.1)	447	348.4–545
Residual effect of previous erythropoiesisstimulating agent dose (RSA)	0.153 (0.7)	0.152	0.149–0.155
Correction factor (CF)	2.75E–4 (0.9)	2.8E–4	2.2E–4–3.3E–4
Slope of age for CF on log–scale	–0.00314 (21.5)	–0.00021	–0.01346 to 0.0144
Slope of ESAD on baseline hemoglobin onlog–scale	–4.49E–7 (54.8)	–4.33E–7	–11.5E–7 to 2.232E–7
ω^2^ on RSA Effect	0.0130 (8.8)	0.0124	0.00766–0.01966
ω^2^ on EC_50_	8.92 (9.5)	9.47	3.61–25.78
ω^2^ on BL Hgb	0.00485 (7.9)	0.00476	0.00393–0.00563
ω^2^ on CF	10.6 (8.0)	11.6	7.244–20.88
σ^2^ (additive component)	0.00478 (0.4)	0.00475	0.0044–0.00511
IIV in RSA	11.4%	NA	NA
IIV in EC_50_	298.7%	NA	NA
IIV in BL Hgb	7.0%	NA	NA
IIV in CF	325.6%	NA	NA
Residual Variability	0.07 SD		

1. Based on 935/1000 successfully converged bootstrap runs;

SD: Standard deviation; NA: not applicable.

Previous ESA dose (ESAD) was identified as a statistically significant predictor of hemoglobin level at baseline and age was identified as a statistically significant predictor of the CF (equations 16 and 17):
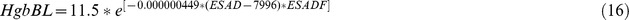


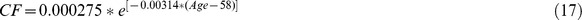



Where:

HgbBL = Model predicted hemoglobin at baseline,

ESADF = an indicator variable with a value of 0 if ESAD = 0 and 1 if ESAD is >0, and

CF = correction factor.

In other words no effect of ESAD was incorporated for subjects whose ESAD dose information was not available.

Inclusion of statistically significant covariates reduced the IIV for RSA and EC50 by 12.6% and 211.3% while the IIV in hemoglobin at baseline was essentially unchanged in the final model, and increased by 11% for CF, relative to the base model.

### Model Validation

**Figure 3:**
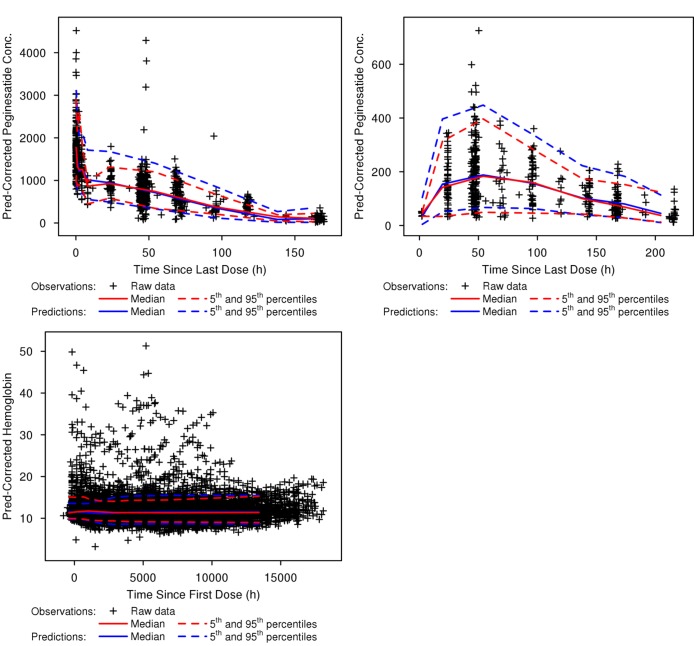
Prediction-corrected visual predictive checks for the final PK model of peginesatide following intravenous and subcutaneous dosing and the final PK-PD model of predicted hemoglobin levels. Upper left: visual predictive check for peginesatide PK model following intravenous administration; Upper right: visual predictive check for peginesatide PK model following subcutaneous administration; Lower panel: visual predictive check for hemoglobin based on final population PK-PD model for peginesatide.

**Figure 4:**
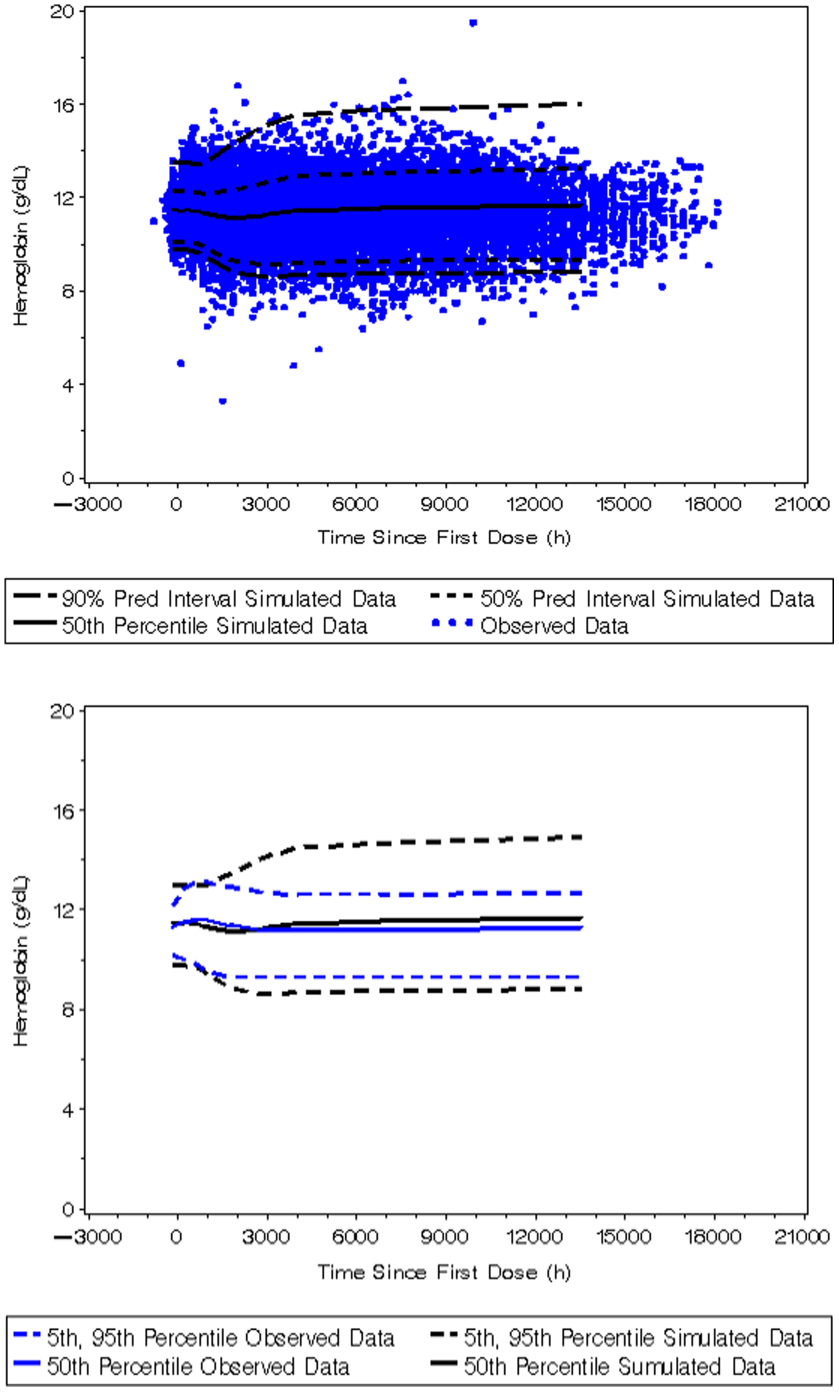
Visual predictive check for the PK-PD model of peginesatide using traditional visual predictive check.

The parameter estimates from the final PK and PK-PD models were also consistent with those estimated from bootstrap evaluations and were within the 95% Confidence Interval (CI) of the median bootstrap estimate, which supports the robustness and stability of the model (**Table 6** and **Table 7**). The results of the pcVPC supported the predictive ability of the PK and PK-PD models, whereby the simulated 90% prediction intervals encompassed approximately 90.9% and 95.2% of the observed peginesatide and hemoglobin concentrations (**Figure 3**), respectively. The pcVPC approach successfully accounted for an inflated estimate of overall variability for the upper 95th percentile following traditional VPCs (**Figure 4**) due to continuous dose adjustment based on hemoglobin response.

### Impact of Covariates on PK and PD Parameters of Peginesatide and Hemoglobin Levels

**Figure 5:**
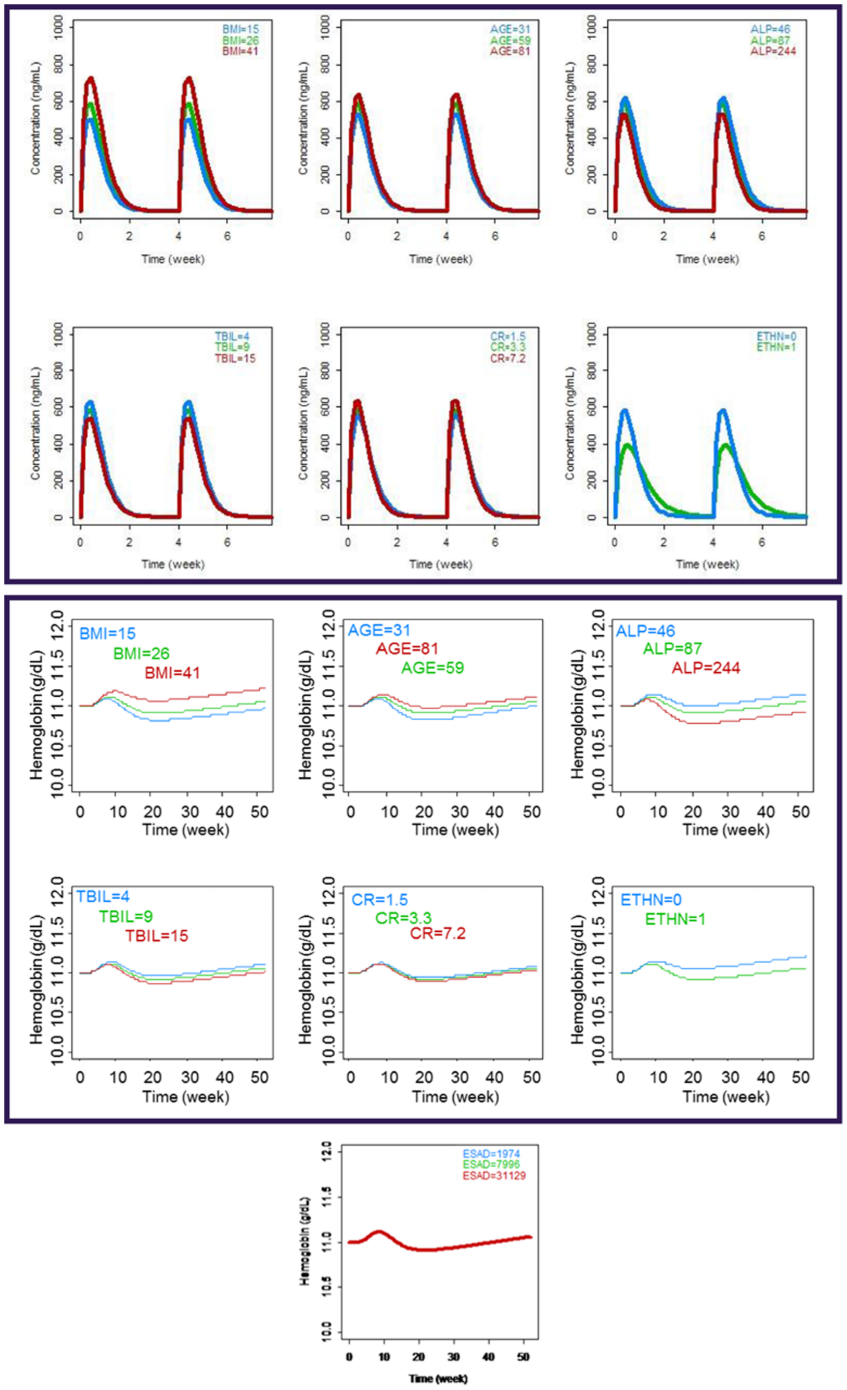
Effect of PK covariates on simulated peginesatide plasma concentrations and hemoglobin levels in patients with chronic kidney disease on dialysis following 10 mg SC dose every 4 weeks for 52 weeks. Upper panel: effect of PK covariates on simulated peginesatide plasma concentrations; Middle and lower panels: effect of PK covariates on simulated peginesatide hemoglobin concentrations. Note that in the panel for hemoglobin levels by ESAD, the effect is very low and, as such, the profiles overlap. Abbreviations: BMI = body mass index, ALP = alkaline phosphatase, TBIL = total bilirubin, CR = serum creatinine, ETHN = ethnicity, and ESAD = erythropoiesis-stimulating agent dose.

The impact of covariates on the PK and PD population mean simulated concentration-time profiles for peginesatide are presented in **Figure 5**. The continuous covariates that were identified as statistically significant for PK, BMI had the largest impact on exposure of peginesatide. Relative to the typical subject with a median BMI of 26 kg/m^2^ and median TBIL, CR and ALP levels of 9.0 g/L, 3.31 mg/dL (in non-dialysis patients) and 87.0 U/L, respectively, subjects experienced up to ±30% change in exposure of peginesatide over the range of BMI evaluated. Effects of all other continuous covariates on peginesatide exposure were less than ±15%. Hispanics exhibited peginesatide Cmax and AUC values approximately 36% and 3% lower, respectively compared to non-Hispanics. The variability in exposure, based on these PK covariates for this population, is predicted to result in a change in hemoglobin levels of ≤0.2 g/dL, following administration of a SC dose of 10 mg given every 4 weeks for 1 year. The effect of significant covariates (ESAD and age) on PD parameters of peginesatide is predicted to result in hemoglobin level changes of <0.05 g/dL. Concomitant medications taken by >10% of the patient population during 80% of the trial period (**Table 2**) did not alter the PK or PD parameters of peginesatide.

## Discussion

The purpose of the current analysis was to develop a population PK-PD model to describe the time-course of peginesatide plasma concentrations and hemoglobin levels following IV and SC administration in patients with CKD. Peginesatide PK parameters were best described by a two-compartment model with first-order absorption and saturable elimination. The relationship between hemoglobin and peginesatide plasma levels was best characterized by a modified precursor-dependent LIDR model.

Based on the population PK model, peginesatide exhibited non-linear pharmacokinetics over the evaluated dose range, which is in agreement with the observed PK profile in healthy volunteers in a Phase I dose-ranging study (unpublished data [AF37702/CPH001]) and consistent with other ESAs [12], [13]. The exact mechanisms for clearance of peginesatide in humans are not known. However elimination of ESAs has been linked with binding to erythropoietin receptor (EPOr) in target tissue, such as bone marrow and spleen. A possible route of elimination could be endocytosis of the EPOr - erythropoietin complex followed by lysosomal degradation as reported by Olsson-Gisleskog et al [14]. The formation of such a complex via receptor binding in the bone marrow may be responsible for the non-linear characteristics observed for peginesatide.

The estimated population value for total volume of distribution (43.0 mL/kg) suggested that tissue distribution was limited following IV and SC administration. The estimated value for the volume of distribution was similar to what observed in healthy volunteers (35 mL/kg). The patient population (i.e., hemodialysis or non-dialysis) did not appear to have a significant effect on peginesatide PK parameters, which may be attributed to the limited amount of non-dialysis patient data (<11%) used in this population PK model. Impact of dialysis on drug levels was not evaluated in this analysis as in the Phase 3 study [9] (which comprised the majority of the data used for PK analysis [81.7%]); only one sample per patient was collected immediately following first dose when subjects were not on dialysis.

**Figure 6:**
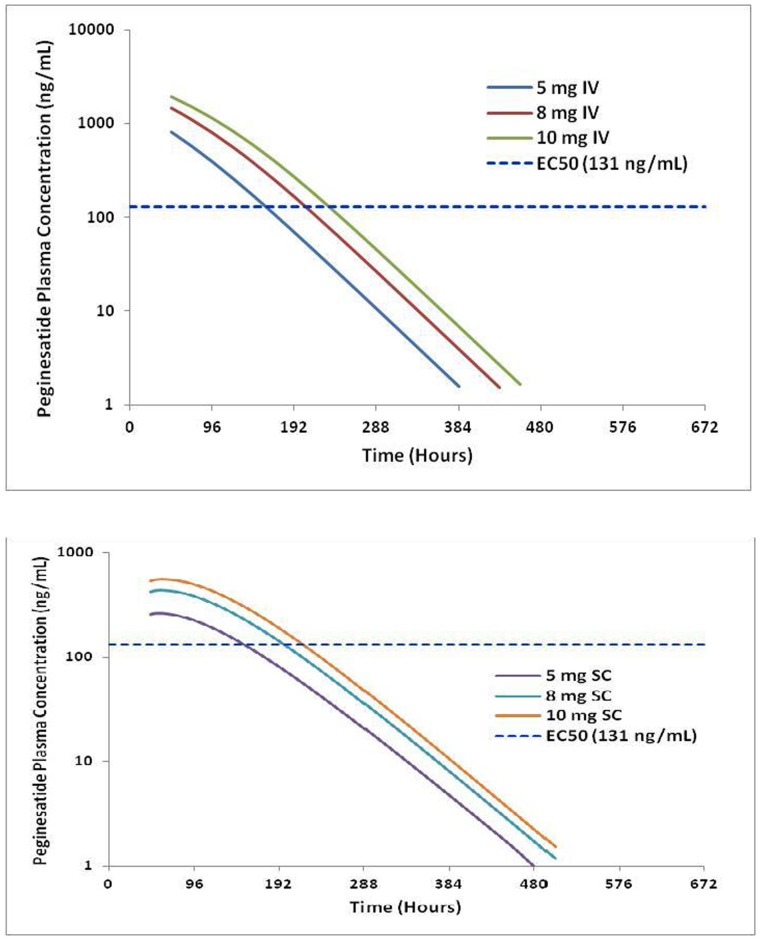
Simulated concentration-time profile of peginesatide following administration of a single 5, 8, and 10 mg every 4 week IV (top panel) and SC (bottom panel) dose. EC50 values reflect the EC50 value estimated for the base PK-PD model.

Peginesatide administered SC has a model estimated bioavailability of 49.8%, which is similar to the range of 33% to 45% reported in studies conducted in healthy volunteers (unpublished data [AFX01_102, AFX01_103) as well as values reported for other ESAs [11]–[15]. Peginesatide, like other erythropoietins, appears to follow flip-flop pharmacokinetics [16] where the rate of absorption is slower than the rate of elimination. Following intravenous administration, peginesatide concentrations exceed the estimated EC50 (concentration required to obtain 50% of the maximum effect for hemoglobin) value, but subsequently decline, rapidly to values below the EC50 during the dosing interval. Following subcutaneous administration, the peak plasma concentrations of peginesatide are lower than those following intravenous administration; however, the plasma concentrations following subcutaneous administration remain sustained above the EC50 for a duration that is comparable to that of intravenous administration. Although the fraction of the dose absorbed from the extravascular compartment following subcutaneous dosing is approximately 50% that of intravenous dosing, subcutaneous dosing provides a similar hemoglobin response. This is further supported by the simulations performed using the final PK model as presented in **Figure 6** where the time above EC50 was similar following IC and SQ every 4 weeks dosing of peginesatide.

BMI, age, ALP, CR (for nondialysis subjects) and TBIL were identified as statistically significant PK covariates. The effect of BMI was included in the final model to account for the variability that is not accounted following weight-normalized dosing of peginesatide. The effect of CR on Ka is attributed to the level of overall patient hydration that may in turn affect the SC absorption of peginesatide. None of the covariates identified are considered clinically relevant based upon their effect on exposure (<36%) of peginesatide and simulated hemoglobin levels (≤0.2 g/dL). The effect of ethnicity was also not considered clinically relevant and, further, may have been confounded by the study site data, with the majority of data from Hispanic subjects (84%) coming from a single clinical site. Concomitant medications did not impact the PK or PD parameters of peginesatide further supporting a lack of potential for drug-drug interactions.

The precursor-dependent LIDR model that described the time-course of peginesatide plasma and hemoglobin levels following IV and SC dosing is similar to the one used for darbepoetin [6]. This model has been used to characterize the effects of hematopoietic growth factors on the proliferation of precursor cells, which mature to circulating red blood cells and eventually die due to apoptosis or random destruction. The small bias observed in the diagnostic plot (observed versus population predicted hemoglobin level) was similar to that observed with the PK-PD analysis conducted for other ESAs [6], [13]. This observed bias may be attributed to the nature of the clinical trials for ESA and peginesatide that included continuous dose adjustment based on the hemoglobin levels.

The estimates of red blood cell life span (67.5 days) obtained from this analysis are consistent with previously reported values in the literature (60 to 65 days in dialysis patients) [15], [17]. The estimated EC50 for peginesatide was higher compared to darbepoetin alfa (0.41 ng/mL), Micera (0.898 ng/mL) and peptidic erythropoiesis receptor agonist (ERA) (120 ng/mL) and associated with a high degree of IIV (298.6% CV), similar to observations of pegylated epoetin beta (559% CV) [6], [13], [18]. The estimated value for the Emax for peginesatide was similar to the values observed for darbepoetin (0.637), Micera (0.425) and ERA (1.68) [6], [13], [18]. The differences in EC50 and Emax observed across the ESAs to some extent is attributed to the fact that estimates were derived using data obtained from different populations (dialysis versus non dialysis) and analyzed using different structural models or might actually reflect differences in binding ability of the molecule to erythropoeitin receptors. The higher EC50 value of peginesatide is also expected due to pegylation of the peptide moiety that binds with the erythropoietin receptors. A CF was empirically derived in the model to account for the potential downward drift in hemoglobin levels observed in patients over the course of the trial period and was found to improve the model fit. However, no placebo data in subjects with CKD were available. Thus, the CF could not be correlated to the rate of change in hemoglobin levels due to progression of the underlying disease in this patient population in the absence of therapeutic intervention.

The pcVPCs of the PK-PD model showed that the simulated prediction intervals were in close agreement with the corresponding percentiles of the observed data. The standard VPC of the PK-PD model (**Figure 4**) showed significant bias in the upper bound of the 90% prediction interval. This is likely due to the very high degree of IIV associated with the estimation of EC50 and individualized dose adjustment implemented in the phase 3 trial based upon observed hemoglobin level. As a result, model predictions should be limited to the evaluated hemodialysis population and additional bias may be observed if the model is extrapolated beyond the evaluated conditions. The traditional VPC has been shown to be uninformative with regard to assessing and diagnosing possible model misspecifications when an adaptive design such as an a posteriori dose adaptation scheme is implemented during a trial [11]. As demonstrated in this case, the use of prediction correction during the VPC provides a more appropriate evaluation of model predictability.

Subjects on lower doses of ESA at baseline generally had higher baseline hemoglobin values compared to those on higher doses. This may be attributed to the fact that patients on a higher ESA dose prior to peginesatide treatment were relatively less sensitive to previous ESA treatment and may reflect poor responders to ESA treatment due various factors [19].

In conclusion, this modeling effort supported the characterization of the PK and PK-PD relationship for peginesatide in subjects with CKD on hemodialysis. Further, although some covariates were found to be statistically significant predictors of variability, their impact on the PK and PD of peginesatide was not clinically relevant and therefore, no dosage adjustments based on patient characteristics or concomitant medications are indicated.
